# *Gryllus bimaculatus* Hydrolysate Ameliorates Obesity-Induced Muscle Atrophy by Activating Skeletal Muscle AMPK in Mice

**DOI:** 10.3390/nu17121990

**Published:** 2025-06-12

**Authors:** Kyungeun Park, Sunyoon Jung, Chunmei Li, Jung-Heun Ha, Yoonhwa Jeong

**Affiliations:** 1 Department of Food Science and Nutrition, Dankook University, Cheonan 31116, Republic of Korea; 2 Research Center for Industrialization of Natural Neutralization, Dankook University, Yongin 16890, Republic of Korea; 3 College of Tourism and Cuisine, Yangzhou University, Yangzhou 225127, China; 4 Department of Culinary Science, Ministry of Culture & Tourism, Yangzhou 225127, China

**Keywords:** AMPK, *Gryllus bimaculatus*, insulin resistance, skeletal muscle, obesity, ectopic fat accumulation

## Abstract

**Background/Objectives**: Obesity-related metabolic complications contribute to musculoskeletal disorders and are often associated with muscular fat accumulation. The AMP-activated protein kinase (AMPK) is a therapeutic target that can mitigate these effects. **Methods**: An *in vivo* study was conducted to understand the effects of *Gryllus bimaculatus* (GB), a potent AMPK activator, on metabolic and muscular homeostasis in diet-induced obesity (DIO). Six-week-old male C57BL/6J mice were fed either a normal diet or a high-fat diet (HFD) for eight weeks to induce DIO. Subsequently, HFD-fed mice were divided into four groups: HFD only, HFD with 100 mg/kg/day GB, HFD with 200 mg/kg/day GB, and HFD with 400 mg/kg/day GB for 16 weeks. To assess the effects of GB, we evaluated insulin resistance, muscle strength, muscular fat accumulation, and AMPK activation using an oral glucose tolerance test, grip strength test, histological assessments, serum lipid analyses, western blotting, and quantitative reverse transcription–polymerase chain reaction. **Results**: The low- and mid-dose GB groups showed a trend toward improved insulin resistance. GB significantly reduced muscle fat accumulation and increased muscle strength. The mid- and high-dose GB groups showed a significantly upregulated expression of the molecular markers of mitochondrial biogenesis and fatty acid oxidation in muscle tissues. Additionally, the high-dose GB group activated AMPK and inhibited the activity of acetyl-CoA carboxylase in the skeletal muscle. **Conclusions**: The results suggest that GB may serve as a nutraceutical candidate for the management of obesity-associated metabolic complications.

## 1. Introduction

Obesity is a metabolic complication whose incidence has steadily increased in recent decades. By 2035, over 50% of the world’s population will be considered obese or overweight [1]. Furthermore, medical costs for the prevention and treatment of obesity are expected to exceed USD 4 trillion per year globally [2]. Obesity is characterized by the excessive accumulation of white adipose tissue (WAT), which occurs when energy intake exceeds energy expenditure [3]. The overconsumption of an extra-fat is considered a major culprit in the development of obesity [4]. In individuals with obesity, excess WAT is located in the visceral and subcutaneous parts of the body in the form of triglycerides (TG) [5]. WAT plays a notable role in energy storage, protection, insulation, and endocrine function [6]. Obesity is a major risk factor for developing metabolic complications, including insulin resistance (IR), glucose intolerance, type 2 diabetes mellitus (T2D), dyslipidemia, and hypertension [3]. Ectopic fat in the skeletal muscle is an important pathological trigger for metabolic complications, including IR [7]. Furthermore, an excessive accumulation of WAT ultimately leads to the infiltration of ectopic fat in metabolic organs, such as the skeletal muscle, liver, and pancreas [4]. Ectopic fat accumulation is closely linked to IR and increases vulnerability to cellular damage [8]. Therefore, prolonged ectopic fat accumulation may increase the risk of oxidative stress, mitochondrial dysfunction, and proinflammatory responses, both locally and systemically.

Ectopic fat accumulation in muscle tissues may deteriorate the innate skeletal muscle function, thereby diminishing physical strength and causing various pathological responses [9,10]. Excessive fat can impair essential skeletal muscle functions, such as insulin-stimulated glucose uptake, fatty acid oxidation (FAO), and mitochondrial functions that regulate energy homeostasis [7,11]. Patients with obesity are likely to develop metabolic dysfunction, including reduced FAO and increased lipid storage in the skeletal muscles [8]. This condition desensitizes insulin signaling in the skeletal muscles and leads to musculoskeletal disorders by inducing mitochondrial dysfunction [9,12,13]. AMPK is a central master regulator of glucose and fatty acid metabolism in skeletal muscles [14]. Structurally, AMPK is a heterotrimer comprising a catalytic subunit (alpha) and two regulatory subunits (beta and gamma) [15]. AMP and ADP bind allosterically to the regulatory γ-subunits of AMPK complex, promoting the phosphorylation of Thr172 residue and triggering the activation of AMPK to its downstream targets [16,17]. Upon activation, AMPK inhibits anabolic pathways, such as lipogenesis, and activates catabolic pathways, including glycolysis and FAO [12,18]. Therefore, AMPK is a potential key therapeutic player in treating various metabolic complications. In addition, AMPK phosphorylation enhances mitochondrial biogenesis, fatty acid uptake and oxidation, leading to increased ATP levels and decreased ATP consumption in different tissues [19]. Therefore, AMPK activation in the skeletal muscles is considered an important therapeutic strategy for combating obesity-associated metabolic complications.

Pharmacological AMPK activators, such as 5-Aminoimidazole-4-carboxamide ribonucleotide (AICAR) and metformin, have been explored for their metabolic effects, including improved insulin sensitivity, lipid metabolism, and mitochondrial function [20]. AICAR, an AMP analog, directly induces AMPK phosphorylation [17] and stimulates FAO and glucose uptake in skeletal muscles [18]. Similarly, metformin, a drug widely prescribed for T2D, activates AMPK by stimulating the phosphorylation of Thr172 on its catalytic subunits [20]. However, metformin has been associated with muscle dysfunction [21], whereas AICAR, which is primarily studied in research settings, has been linked to neurodegeneration [22]. Thus, natural ergogenic compounds that activate AMPK may provide safer alternatives for supporting metabolic health. Natural substances have long been the primary source of drugs for various diseases because of their numerous bioactive properties and few side effects [9,11,23]. Natural products typically have greater chemical diversity and higher binding affinities for specific receptor systems than synthetic molecules [24]. Herbal products are extracts from seeds, gums, roots, pods, flowers, or leaves and contain phytochemicals, such as carotenoids, polyphenols, and tannins, some of which are known to enhance muscle growth and fat burning [11,23]. Among dietary polyphenols, resveratrol is a potent AMPK activator [24,25], improving mitochondrial activity and protecting against metabolic disease caused by diet-induced factors [26,27,28].

In recent years, more than 2000 edible insects have attracted global attention because of their functionality and human consumption functionality [29,30]. The Food and Agriculture Organization has predicted that the world population will increase to 9.1 billion people by 2050 [31]. However, current food supplements are not enough to meet the needs of the future world population, requiring more land and water to farm livestock [32]. Eating edible insects has been suggested as an environmentally sustainable alternative for meat to reduce greenhouse gas emissions, given the reduced ecological impact of edible insect farming [33,34]. For example, crickets require only 2 kg of feed for every 1 kg of body weight gain and can be reared on organic side streams, thereby reducing environmental contamination [35]. Edible insects are a rich supply of essential amino acids, fiber, vitamins, minerals, essential fatty acids (omega-3 and omega-6 fatty acids), in addition to containing large amounts of polyphenols, which play a fundamental role in specific bioactivities [36]. Although notable variations have been found in a wide variety of species, many edible insects provide sufficient amounts of energy and protein to meet the amino acid requirements of humans [37]. Moreover, based on the diversity of plants encountered by phytophagous insects, flavonoids appear to be the most absorbed, which is fundamentally attributed to herbivorous feeding behavior [38]. Thus, these bioactive substances present in edible insects indicate their functional potential and their promising future as natural ergogenic supplements [36,38].

Here, we assessed the potential of *Gryllus bimaculatus* (GB) as a natural, edible, and potent AMPK agonist. GB is approved as an edible insect by the Korea Food and Drug Administration and known for its high nutritional value [39,40]. GB contains high amounts of protein, essential amino acids, and minerals, including phosphorus (P), sodium (Na), and calcium (Ca) [41,42]. Moreover, GB has remarkable biological functions, such as anti-sarcopenic [43,44], anti-inflammatory [45,46,47], and glucose-lowering effects [43,48], because of its multiple bioactive components. Recent studies have demonstrated that GB acts as an AMPK agonist [43]. In rodents, an oral gavage of the GB extract ameliorated HFD-induced hyperglycaemia and hyperlipidaemia by inhibiting hepatic lipogenesis via AMPK activation [43]. In another study using a murine model, GB feeding increased muscle anabolism by activating the Akt/mammalian target of rapamycin (mTOR)/S6K pathway and preventing the activation of dexamethasone-induced muscle atrophic factors [44]. Intriguingly, GB has shown antithetical roles in the two major metabolic organs, liver and muscle, inducing catabolic effects in the liver via AMPK activation [43] and anabolic effects in the skeletal muscle via the Akt/mTOR/S6K pathway [44]. Based on previous studies, we aimed to examine whether GB prevents metabolic complications, such as obesity, glucose intolerance, and muscle dysfunction, in both skeletal muscles and WAT in a DIO murine model.

## 2. Materials and Methods

### 2.1. Hydrolysis of GB Powder Using Enzyme

GB powder underwent enzymatic hydrolysis using a slightly modified version of a previously established method [49]. In summary, defatted GB powder (Hanmi Nutrition Inc., Paju, Republic of Korea) was suspended in distilled water and treated with 1% (*v*/*w*) Alcalase^®^ (Novozymes, Bagsvaerd, Denmark). The hydrolysis was carried out at 60 °C for 5 h continuous shaking using a shaking water bath (MaXturdy 45; Daihan Scientific, Wonju, Republic of Korea). The pH solution was maintained at 8.0 by adding 1N NaOH. To stop the enzymatic reaction, the mixture was heated to 95 °C for 20 min. The resultant then centrifuged at 2000× *g* for 15 min to remove insoluble residual materials. The resulting supernatant was filtered through a 6-μm pore-size filter paper (Advantec, Tokyo, Japan) using a vacuum pump (Gast Manufacturing, Benton Harbor, MI, USA). The filtrate was subsequently freeze-dried and preserved at −70 °C until further use.

### 2.2. Animal Experiments

**Figure 1:**
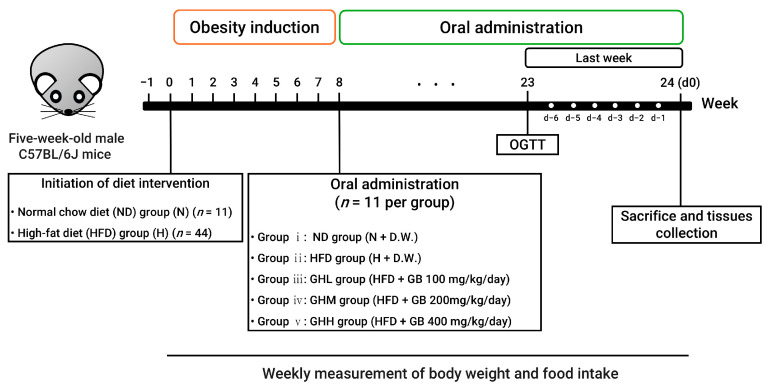
Experimental scheme. d, day; D.W., distilled water; GB, *Gryllus bimaculatus*; GHH, HFD + 400 mg/kg; GHL, HFD + 100 mg/kg GB; GHM, HFD + 200 mg/kg GB; H, high-fat diet group; HFD, high-fat diet; N, normal diet group; ND, normal diet; OGTT, oral glucose tolerance test.

Five-week-old male C57BL/6J mice (Raon Bio, Yongin, Republic of Korea) were maintained housed (2–3 mice/cage) at a controlled room temperature environment (24 ± 2 °C) under a 12 h light–dark cycle with *ad libitum* access to food and water. C57BL/6J mouse strains are widely used for the study of obesity. The sample size was determined to be a minimum of 9 per group to achieve a statistical power 80% with a significance level below 5%, while accounting for potential dropout (2 additional mice) by including 2 additional mice per group. After one week of acclimatization, the mice were randomly assigned to the following experimental groups: (i) a normal diet group (N, 10% kcal from fat; D12450J, Research Diets, New Brunswick, NJ, USA) and a high-fat diet group (H, 60% kcal from fat; D12492, Research Diets, New Brunswick, NJ, USA). The N group was fed a normal diet (ND) or a HFD for two months to induce obesity. After eight weeks, the HFD-fed mice were randomly divided into four subgroups for a 16-week dietary intervention period: (ii) HFD group (H), (iii) HFD + 100 mg/kg/day GB (GHL), (iv) HFD + 200 mg/kg/day GB (GHM), and (v) HFD + 400 mg/kg/day GB (GHH). The N group served solely as a reference to confirm the successful induction of metabolic disturbances by HFD to facilitate investigating the therapeutic potential of GB under conditions of DIO. The GB doses given to the mice were estimated from human-equivalent doses that may have clinical relevance (0.5, 1.0, and 2.0 g/60 kg/day), as referenced in a prior study [49,50]. GB was administered by daily oral gavage for 16 consecutive weeks while continuing with the HFD or ND (Figure 1).

The N and H groups received oral administered of distilled water as a control vehicle. The body weight and food intake were recorded on a weekly basis. After 24 weeks of treatment and a 12 h fasting period, the experimental mice were anesthetized with 50% isoflurane (Hana Pharm., Seoul, Republic of Korea) diluted in propylene glycol (Daejung Chemicals, Siheung, Republic of Korea), then euthanized by cardiac puncture-induced exsanguination. Blood was collected immediately, and skeletal muscle along with WAT was harvested. The collected tissues were either preserved in 10% neutral-buffered formalin (NBF) (Sigma-Aldrich, St. Louis, MO, USA) or rapidly frozen in liquid nitrogen and stored at −70 °C for further analysis. All procedures followed the ethical standards set by the Institutional Animal Care and Use Committee of Dankook University (IACUC No. DKU-22-086).

### 2.3. Serum Biochemistry

Blood samples were obtained and centrifuged at 3000× *g* for 15 min at 4 °C to isolate the serum [51]. Serum levels of TG, total cholesterol (TC), and high-density lipoprotein cholesterol (HDL-C) levels were assessed using commercially available kits (Embiel, Gunpo, Republic of Korea). The concentration of low-density lipoprotein cholesterol (LDL-C) was calculated using the Friedewald formula: LDL-C = TC − HDL-C − (TG/5), in accordance with previously established methods [52]. Liver function markers, including alanine aminotransferase (ALT) and aspartate aminotransferase (AST), were evaluated with enzymatic assay kits (Asan Pharmaceutical Co., Seoul, Republic of Korea). Insulin levels in serum were quantified using a mouse-specific enzyme-linked immunosorbent assay (ELISA) using a mouse insulin ELISA kit (Crystal Chem, Zaandam, The Netherlands), and fasting glucose was measured with a commercial kit (Asan Pharmaceutical Co., Seoul, Republic of Korea).

### 2.4. Grip Strength Test

Grip strength was measured at weeks 14, 15, and 16 after dietary intervention. To assess forelimb strength, a grip strength meter (BioSeb, Chaville, France) was used. Each mouse was positioned at the center of the grid, and gentle traction was applied to the tail in alignment with the grid’s direction. The peak force exerted during five repeated trials was recorded, and the average value was adjusted based on body weight.

### 2.5. Oral Glucose Tolerance Test (OGTT)

To evaluate glucose metabolism, an OGTT was conducted nine days before the animals were sacrificed. Mice were fasted for six hours prior to the test, after which glucose (1 g/kg body weight; Sigma-Aldrich) was orally administered. Blood glucose concentrations were measured from the tail vein at 0 (baseline), 15, 30, 60, and 120 min using a glucometer (Accu-Chek, Roche, Basel, Switzerland). The area under the curve (AUC) for glucose levels was determined using the trapezoidal rule with GraphPad Prism version 9.5 (GraphPad, La Jolla, CA, USA) [53]. Insulin resistance was assessed using the homeostasis model assessment-estimated insulin resistance (HOMA-IR), calculated as fasting insulin (ng/mL) × fasting glucose (mg/dL) ÷ 405.

### 2.6. Histological Analysis

For microscopic evaluation, portions of the gastrocnemius (GA) muscle and epididymal white adipose tissue (EAT) were fixed in 10% neutral-buffered formalin. The fixed samples were embedded in paraffin, sectioned at a thickness of 4 μm, and stained with hematoxylin and eosin (H&E). Histological observations were conducted using an optical microscope (BX53, Olympus, Tokyo, Japan) at 40× magnification. The cross-sectional area (CSA) of muscle fibers and the size of adipocytes were quantified using ImageJ software (version 1.53t, NIH, Bethesda, MD, USA) at three randomly selected fields per slide.

### 2.7. Muscle Lipid Contents

Total lipids from quadriceps muscle were extracted using the method of Folch et al. [54,55]. Briefly, the muscle tissues were homogenized in chloroform–methanol (2:1, *v*/*v*) at a ratio of 1:20 (*w*/*v*) and incubated at 4 °C for one hour. The homogenate was then centrifuged at 2400× *g* for 10 min. The supernatant was combined with 0.88% sodium chloride (0.2 volume) to form a biphasic mixture (chloroform–methanol–water = 8:4:3, *v*/*v*/*v*). Following an additional centrifugation step (15 min, 2400× *g*), the lower organic phase was washed twice with a solution of water–methanol–chloroform (15:16:1, *v*/*v*/*v*). The extracted lipids were air-dried and reconstituted in deionized water. Triglyceride (TG) and total cholesterol (TC) levels were analyzed using colorimetric assay kits (Embiel).

### 2.8. Total RNA Extraction and Quantitative Real Time PCR (qRT-PCR)

qRT-PCR was performed with minor modifications from previously reported protocols [56]. Total RNA was extracted from GA muscle samples using NucleoZOL reagent (Macherey-Nagel, Bethlehem, PA, USA). Complementary DNA (cDNA) synthesis was carried out using RocketScript™ Reverse Transcriptase, RNase H Minus (Bioneer, Daejeon, Republic of Korea) according to the manufacturer’s instructions. Amplification and quantification were performed using a CFX Connect Real-Time PCR System (Bio-Rad, Hercules, CA, USA) and iQ™ SYBR^®^ Green Supermix (Bio-Rad). Gene expression was normalized to ribosomal protein lateral stalk subunit P0 (*Rplp0*, also known as *36b4*). The relative expression levels were calculated using the 2^−ΔΔCT^ method [57] and presented as fold changes relative to the H group. Primer sequences are detailed in Table S1.

### 2.9. Western Blot Analysis

GA muscle tissues were lysed in ice-cold RIPA buffer (Elpis Biotech, Daejeon, Republic of Korea) supplemented with protease and phosphatase inhibitors (Roche Applied Science, Penzberg, Germany). Lysates were centrifuged at 12,000× *g* for 20 min at 4 °C to collect the protein-containing supernatants. Protein concentrations were measured using a BCA protein assay kit (Thermo Scientific, Waltham, MA, USA). Equal amounts of protein (20–30 μg) were loaded onto 10% SDS-PAGE gels, separated, and transferred to polyvinylidene difluoride (PVDF) membranes (Bio-Rad) at 100 V for 1 h. Membranes were blocked with 5% skim milk (Difco, Detroit, MI, USA) for 1 h at room temperature and incubated overnight at 4 °C with primary antibodies. After washing with Tris-buffered saline containing 0.1% Tween 20, membranes were incubated with secondary antibodies for 1 h at room temperature. Antibodies used are listed in Table S2. Protein bands were detected using a chemiluminescence system and visualized with the iBright CL750 imaging platform (Invitrogen, Carlsbad, CA, USA). Band intensities were quantified using iBright Analysis Software (version 5.2.1, Invitrogen).

### 2.10. Statistical Analysis

All values are expressed as the means ± standard error of the mean (SEM). Group comparisons were conducted using one-way analysis of variance (ANOVA), followed by Tukey’s multiple comparison test. For nonparametric factorial data, the aligned rank transform (ART) was applied to convert the values into ranks prior to ANOVA, which relies on a preprocessing step that aligns data before applying the averaged ranks, thereby making it possible to use ANOVA procedures [58]. Outliers were excluded using the robust regression and outlier removal method. Statistical significance was set at *p* < 0.05. Analyses were performed using GraphPad Prism 9.5 (GraphPad).

## 3. Results

### 3.1. Effect of GB Supplementation on Body Weight Changes, Energy Intake, and Fat Mass

**Figure 2:**
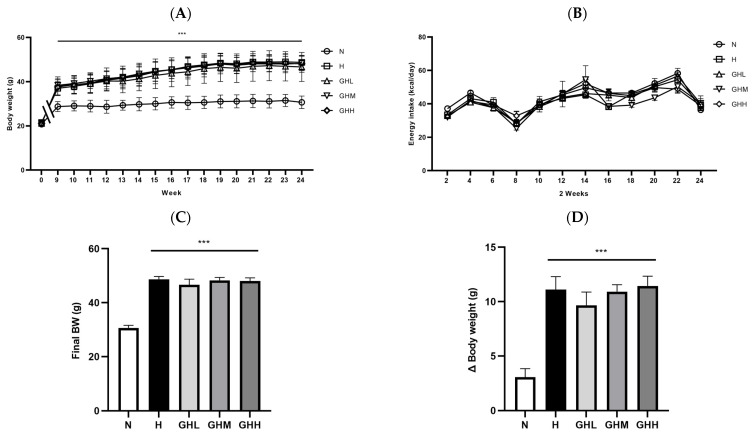

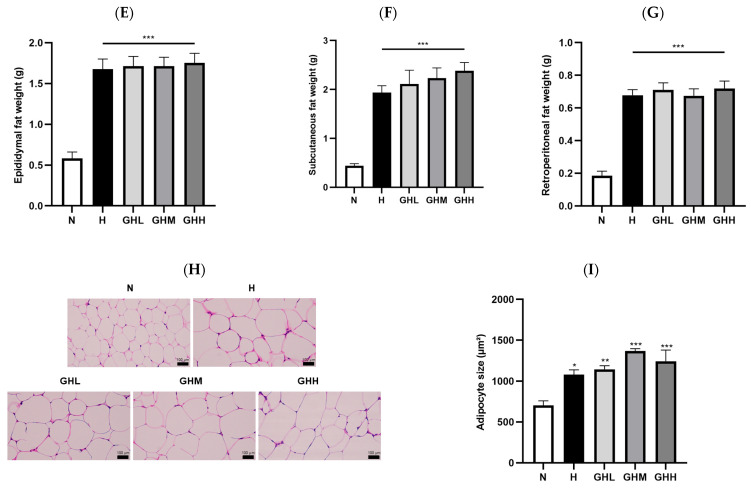
The effect of GB on obesity in DIO mice. C57BL/6J mice were fed an HFD for eight weeks to induce obesity. GB was administered for 16 weeks with HFD feeding. (**A**) Weekly change in body weight (*n* = 9–11). (**B**) Energy intake (*n* = 9–11). (**C**) Final body weight (*n* = 9–11). (**D**) Weight gain (*n* = 9–11). Fat mass of (**E**) epididymal white adipose tissue (EAT) (*n* = 9–11), (**F**) subcutaneous white adipose tissue (SQT) (*n* = 9–11), and (**G**) retroperitoneal white adipose tissue (RAT) (*n* = 9–11). (**H**) H&E staining and (**I**) cell size of EAT (*n* = 9–11). No significant differences were noted among the H groups (H, GHL, GHM, and GHH). Data represent the means ± SEM. * < 0.05, ** < 0.01, *** < 0.001 versus N group. BW, body weight; GB, *Gryllus bimaculatus*; GHH, HFD + 400 mg/kg; GHL, HFD + 100 mg/kg GB; GHM, HFD + 200 mg/kg GB; H, high-fat diet group; HFD, high-fat diet; N, normal diet group.

To determine whether GB supplementation prevents metabolic complications in a DIO murine model, six-week-old C57BL/6J mice were fed an HFD for 8 weeks as a lead-in period (Figure 1). GB was administered at three different dosages for an additional 16 consecutive weeks while continuing the HFD (Figure 1). After the initial 8 weeks, body weight in the H group was elevated by 25% compared to the N group, with the difference reaching statistical significance (*p* = 0.0008) (Figure 2A). Thus, we confirmed that our experimental protocol was suitable for inducing a DIO murine model to investigate obesity-related metabolic disorders prior to GB administration. During the subsequent 16 weeks of GB administration, body weight steadily increased in the H group; however, GB supplementation did not affect weekly changes in body weight (Figure 2A). No changes in energy intake were observed following GB supplementation (Figure 2B). At the end of the experiment, the H group showed significantly increased final body weight and body weight gain by 59% and 72%, respectively, compared to mice in the N group (*p* < 0.0001) (Figure 2C,D). However, GB supplementation did not affect final body weight or body weight gain (Figure 2C,D). The consistent body weight gain among the H- and GB-supplemented groups may be due to unaltered energy intake during the dietary intervention period (Figure 2B). Furthermore, the masses of EAT, subcutaneous white adipose tissue (SQT), and retroperitoneal white adipose tissue (RAT) were significantly higher in all HFD-fed groups than in the N group, regardless of GB supplementation (*p* < 0.0001) (Figure 2E–G). Additionally, adipocyte size was significantly increased in the HFD-fed groups relative to that in the N group (*p* = 0.0150) (Figure 2H,I). These results indicate that GB supplementation did not prevent body weight and WAT gain associated with HFD consumption.

### 3.2. Effect of GB Supplementation on Glucose Homeostasis

**Figure 3:**
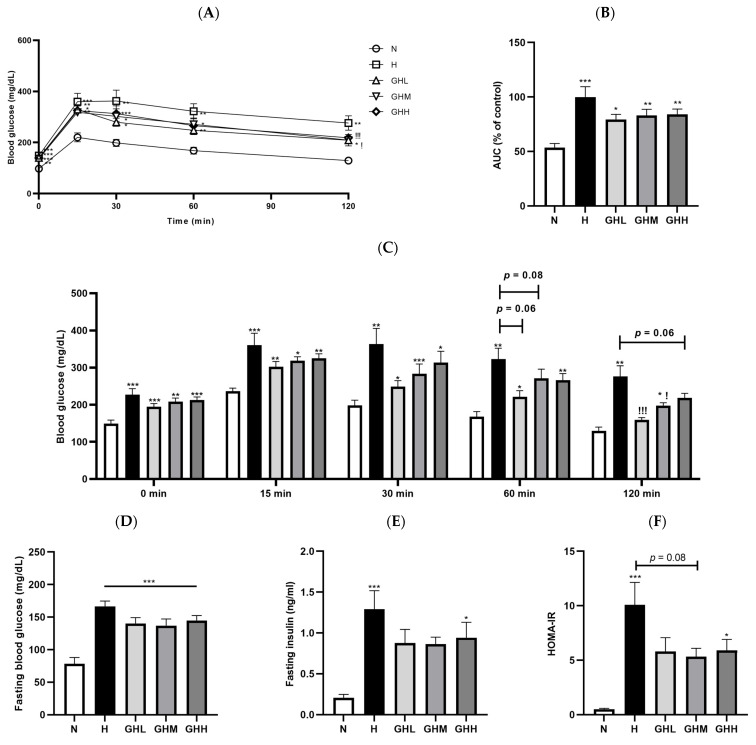
The effects of GB on glucose homeostasis in DIO mice. (**A**) Oral glucose tolerance test (OGTT) at week 15 of GB administration (*n* = 9–11). (**B**) Area under the curve (AUC) (*n* = 9–11) and (**C**) time-based blood glucose levels from the OGTT curve (*n* = 9–11). (**D**) Fasting blood glucose level in serum at the time of sacrifice (*n* = 9–11). (**E**) Fasting insulin level in serum at the time of sacrifice (*n* = 8–11). (**F**) Serum homeostasis model assessment-estimated insulin resistance (HOMA-IR) at the time of sacrifice (*n* = 7–11). Data represent the means ± SEM. * < 0.05, ** < 0.01, *** < 0.001 versus N group. ! < 0.05, !!! < 0.001 versus H group. AUC, area under the curve; GB, *Gryllus bimaculatus*; GHH, HFD + 400 mg/kg; GHL, HFD + 100 mg/kg GB; GHM, HFD + 200 mg/kg GB; H, high-fat diet group; HFD, high-fat diet; N, normal diet group.

To determine whether GB administration affected glucose homeostasis, an OGTT was conducted after 15 weeks of dietary intervention. The H group had higher blood glucose levels than the N group (Figure 3A). The AUC of blood glucose levels was significantly higher in the H group than that in the N group (*p* < 0.0001); however, GB supplementation partially prevented this increase, reducing the AUC by 21%, 17%, and 16% with GHL, GHM, and GHH supplementation, respectively, although the difference was not statistically significant (Figure 3B). Consistent with previously reported data, GB administration ameliorated glucose intolerance at 60 min in the GHL (*p* = 0.0608) and GHM (*p* = 0.0886) groups and showed a tendency toward lower blood glucose levels, although this difference was not significant (Figure 3C). GB supplementation resulted in significantly lower blood glucose levels at 120 min after oral administration of the glucose solution in the GHL (*p* = 0.0010) and GHM (*p* = 0.0110) groups (Figure 3C). Fasting blood glucose levels at the time of euthanasia were higher in the H group than in the N group (*p* < 0.0001). However, the GHL (~16%) and GHM (~17%) groups exhibited lower blood glucose levels than the H group, although the differences were not statistically significant (Figure 3D). In addition, HFD supplementation reduced insulin sensitivity regardless of GB consumption (*p* < 0.0001) (Figure 3E). However, serum insulin levels were reduced in HFD-fed mice supplemented with GB for 16 weeks by 32% and 33% at low- and mid-doses of GB, respectively (Figure 3E). Furthermore, HOMA-IR was lower in the H group than in the N group (*p* = 0.0001); however, GB supplementation enhanced insulin sensitivity by 42%, 47%, and 41% in the GHL, GHM, and GHH groups, respectively (Figure 3F). Therefore, our data indicated that GB supplementation partially improved glucose tolerance and insulin sensitivity in mice with DIO.

### 3.3. Effects of GB Supplementation on Muscle Function and Muscular Ectopic Fat Accumulation

GB supplementation had significant preventive effects on HFD-inducible glucose intolerance, albeit only partially. Therefore, we postulated that GB supplementation might prevent hyperlipidaemia and/or hypercholesterolaemia in experimental mice. GB supplementation did not alter the lipid panel compared to the H group (Table 1). Lipid panel components such as serum TC (*p* = 0.0216) and LDL-C (*p* = 0.0215) were significantly higher in the H group than in the N group; however, no preventive effects on the lipid panel were observed in any of the GB-supplemented groups (Table 1).

**Figure 4:**
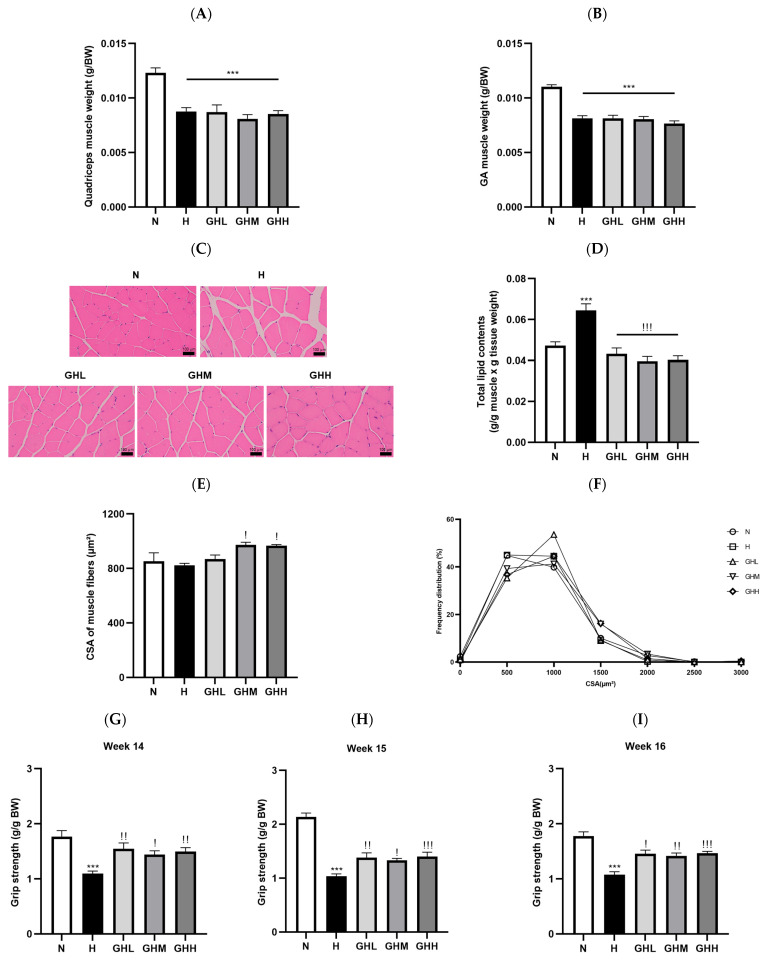
The effects of GB on muscle function and ectopic fat accumulation. Ratio of muscle mass of (**A**) quadriceps (*n* = 9–11) and (**B**) gastrocnemius (GA) to body weight (*n* = 7–11). (**C**) H&E staining of GA muscle. (**D**) Total lipid contents of quadriceps muscle (*n* = 9–11). (**E**) Cross-sectional area (CSA) of muscle fibers (*n* = 9–11). (**F**) CSA distribution of the GA muscle fibers (*n* = 9–11). Muscle function was measured based on grip strength at weeks (**G**) 14 (*n* = 9–11), (**H**) 15 (*n* = 8–11), and (**I**) 16 (*n* = 9–11) after GB administration. The results in (**E**,**G**,**H**) were statistically analyzed using the aligned rank transform (ART) method. Data represent the means ± SEM. *** < 0.001 versus N group. ! < 0.05, !! < 0.01, !!! < 0.001 versus H group. BW, body weight; CSA, cross-sectional area; GA, gastrocnemius; GB, *Gryllus bimaculatus*; GHH, HFD + 400 mg/kg; GHL, HFD + 100 mg/kg GB; GHM, HFD + 200 mg/kg GB; H, high-fat diet group; HFD, high-fat diet; N, normal diet group.

Therefore, we extended our research hypothesis to investigate whether GB supplementation prevents ectopic fat accumulation in the quadriceps, a key organ involved in metabolic responses. Several studies have emphasized the close relationship between ectopic fat accumulation and the development of IR in patients with T2D [59]. The ratio of quadriceps muscle mass to body weight was significantly lower in all HFD-fed groups than in the N group, regardless of GB supplementation (*p* < 0.0001) (Figure 4A). The reduction in the ratio of GA mass to body weight in the H group (*p* < 0.0001) remained unchanged following GB supplementation (Figure 4B). Histological observations using H&E staining and the analysis of muscular lipid content indicated that muscular ectopic fat accumulation in the H group was markedly higher than that in the N group (*p* = 0.0002) (Figure 4C). Intriguingly, after 16 weeks of GB supplementation, a modest reduction in ectopic fat accumulation in the muscle was observed (Figure 4C), along with a decrease in total lipid content in the muscle (*p* < 0.0001) (Figure 4D). The CSA of the muscle fibers was significantly higher in the GHM (*p* = 0.0170) and GHH (*p* = 0.0452) groups than in the H group, which was associated with a reduction in ectopic fat accumulation in the muscle (Figure 4E). Consistent with the CSA of the muscle fibers, the distribution of CSA in the H group was comparable to that in the N group (Figure 4F). However, in the GHL group, muscle fibers with areas of 500–1500 μm^2^ were more frequently observed than in the H group (Figure 4F). To understand how GB supplementation affects muscular functionality, a grip strength test was conducted consecutively at weeks 14, 15, and 16 after dietary intervention. The H group exhibited the lowest grip strength among all experimental groups (*p* < 0.0001); however, the HFD-induced loss of grip strength was prevented in all GB groups (14 weeks: *p* = 0.0026 for GHL, *p* = 0.0188 for GHM, *p* = 0.0037 for GHH; 15 weeks: *p* = 0.0021, *p* = 0.0162, *p* = 0.0005; 16 weeks: *p* = 0.0002, *p* = 0.0012, *p* < 0.0001) (Figure 4G–I). Collectively, these observations in the experimental mice suggest that GB supplementation significantly reduced muscular ectopic fat deposition, increased muscle fiber size, and enhanced muscle strength in DIO mice.

### 3.4. Effects of GB Supplementation on Muscular Ampk Activation

**Figure 5:**
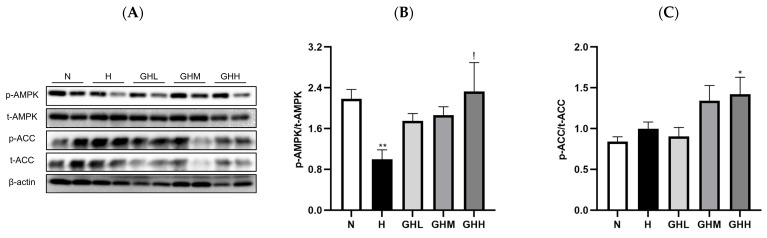
The effects of GB on muscular AMPK activation. (**A**) A representative band image of western blot and the quantification of (**B**) phosphorylated AMPK (*n* = 4), (**C**) phosphorylated ACC (*n* = 9–11). The results in (**B**) were statistically analyzed using the aligned rank transform (ART) method. Data represent the means ± SEM. * < 0.05, ** < 0.01 versus N group. ! < 0.05 versus H group. ACC, acetyl-CoA carboxylase; AMPK, AMP-activated protein kinase; GB, *Gryllus bimaculatus*; GHH, HFD + 400 mg/kg; GHL, HFD + 100 mg/kg GB; GHM, HFD + 200 mg/kg GB; H, high-fat diet group; HFD, high-fat diet; N, normal diet group.

Because of the observed maintenance of muscle function with GB supplementation against DIO, we logically extended our research interest to investigate whether GB supplementation alters the expression of metabolic proteins such as AMPK and acetyl-CoA carboxylase (ACC) in muscles. The activation of the AMPK signaling pathway leads to increased catabolic responses, such as FAO, in skeletal muscles by phosphorylating and inhibiting anabolic responses, such as ACC, thereby promoting muscular fatty acid uptake [18] and inhibiting lipid synthesis [11]. Additionally, the activation of AMPK enhances insulin responsiveness by promoting the movement of intracellular vesicles containing glucose transporter 4 (GLUT4) to the plasma membrane, enabling glucose uptake through carrier-mediated diffusion [9]. In the skeletal muscle of DIO mice, HFD consumption significantly decreased the phosphorylation levels of AMPK by 54% compared to those in the N group (*p* = 0.0079) (Figure 5A,B). However, the GB-supplemented mice showed a prevention of the loss of *p*-AMPK, with GHL, GHM, and GHH (*p* = 0.0364) exhibiting 75%, 86%, and 133% higher relative *p*-AMPK levels, respectively, than the H group (Figure 5A,B). Similarly, HFD consumption did not significantly affect the phosphorylation levels of ACC in the N group; however, GB supplementation prevented the loss of *p*-ACC, with GHM and GHH showing 38% and 46% higher levels, respectively, than in the H group (Figure 5A–C). The results indicate that GB supplementation may activate AMPK, which in turn could suppress the phosphorylation of ACC in skeletal muscle.

### 3.5. Effects of GB Supplementation on Gene and Protein Expression in Skeletal Muscle

**Figure 6:**
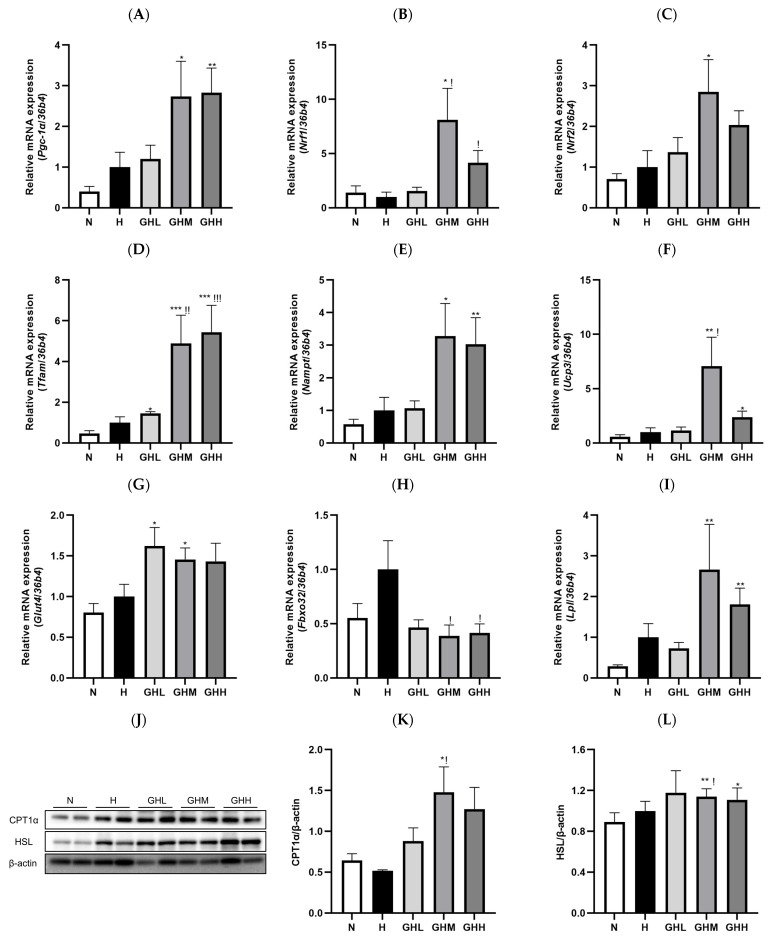
The effects of GB on gene and protein expression in skeletal muscle. The mRNA expression in gastrocnemius muscle; (**A**) *Pgc-1α* (*n* = 7–9), (**B**) *Nrf1* (*n* = 7–9), (**C**) *Nrf2* (*n* = 7–9), (**D**) *Tfam* (*n* = 7–10), (**E**) *Nampt* (*n* = 7–10), (**F**) *Ucp3* (*n* = 7–9), (**G**) *Glut4* (*n* = 9–10), (**H**) *Fbxo32* (*n* = 8–11), and (**I**) *Lpl* (*n* = 8–9). (**J**) Representative band image of western blot and quantification of (**K**) CPT1α (*n* = 9–11) and (**L**) HSL (*n* = 9–11). The results in (**A**,**B**,**D**–**G**,**I**,**L**) were statistically analyzed using the aligned rank transform (ART) method. Data represent the means ± SEM. * < 0.05, ** < 0.01, *** < 0.001 versus N group. ! < 0.05, !! < 0.01, !!! < 0.001 versus H group. CPT1α, carnitine palmitoyltransferase I alpha; Fbxo32, F-box protein 32; GB, *Gryllus bimaculatus*; Glut4, glucose transporter type 4; GHH, HFD + 400 mg/kg; GHL, HFD + 100 mg/kg GB; GHM, HFD + 200 mg/kg GB; H, high-fat diet group; HFD, high-fat diet; HSL, hormone-sensitive lipase; LPL, lipoprotein lipase; N, normal diet group; Nampt, *N*-nicotinamide phosphoribosyltransferase; Nrf, nuclear respiratory factor; Pgc-1α, peroxisome proliferator–activated receptor gamma coactivator-1 alpha; Tfam, mitochondrial transcription factor A; Ucp3, uncoupling protein 3.

To explore the downstream consequences of AMPK activation, we examined the impact of GB on mitochondrial biogenesis and fatty acid oxidation (FAO). Skeletal muscle mitochondria are essential for maintaining cellular energy balance, and their dysfunction has been linked to reduced insulin sensitivity and the development of type 2 diabetes (T2D) [13]. The administration of GB resulted in notable alterations in the expression of key genes involved in mitochondrial function and biogenesis (Figure 6). Peroxisome proliferator-activated receptor gamma coactivator-1 alpha (*Pgc-1α*), a key effector downstream of AMPK and a central regulator of mitochondrial biogenesis, exhibited a marked increase of 173% and 183% in the GHM and GHH groups, respectively, compared to the H group (Figure 6A). In line with this upregulation, the expression levels of nuclear respiratory factor 1 (*Nrf1*) (Figure 6B), nuclear respiratory factor 2 (*Nrf2*) (Figure 6C), and mitochondrial transcription factor A (*Tfam*) (Figure 6D) were also elevated in the skeletal muscle of GB-treated DIO mice. PGC-1α functions as a transcriptional coactivator for NRF1 and NRF2, which subsequently enhance the transcription of mitochondrial genes such as TFAM [26]. Our study showed that *Nrf1* expression increased significantly in the GHM (*p* = 0.0111) and GHH (*p* = 0.0334) groups by 711% and 314%, respectively (Figure 6B). Additionally, *Nrf2* levels increased by 185% and 103% in the GHM and GHH groups, respectively (Figure 6C). Consistent with these findings, *Tfam* gene expression in the skeletal muscle of GB-supplemented DIO mice showed a substantial elevation, increasing by 388% in the GHM group (*p* = 0.0028) and 444% in the GHH group (*p* = 0.0007) (Figure 6D). The activation of AMPK increases intracellular NAD^+^ concentrations through the upregulation of nicotinamide phosphoribosyltransferase (NAMPT), which subsequently enhances sirtuin 1 (SIRT1)-dependent deacetylation and activation of PGC1α. This activation facilitates mitochondrial biogenesis through the involvement of NRF1 and NRF2 [52,60]. During the 16-week GB administration period, *Nampt* gene expression was upregulated by 228% and 203% in the GHM and GHH groups, respectively, compared to that in the HFD-fed group (Figure 6E). Moreover, uncoupling protein 3 (*Ucp3*), a marker of FAO, was significantly upregulated by 607% in the GHM group (*p* = 0.0336) (Figure 6F). Additionally, *Glut4* gene expression was upregulated by 62%, 46%, and 43% with low, medium, and high doses of GB, respectively (Figure 6G), which is consistent with our results showing enhanced insulin sensitivity (Figure 3). The atrogene F-box protein 32 (*Fbxo32*), known to be transcriptionally increased in skeletal muscles under atrophy-inducing conditions, such as obesity [61], was significantly decreased by 61% and 59% with mid- (*p* = 0.0424) and high-dose (*p* = 0.0415) GB administration, respectively, compared to the H group (Figure 6H). Lipoprotein lipase (*Lpl*), a central enzyme in lipid metabolism, also showed an increasing tendency of 166% and 81% in the GHM and GHH groups, respectively, compared to the H group (Figure 6I). FAO serves as the primary mechanism for breaking down fatty acids and supplying energy to cells [49]. Carnitine palmitoyltransferase 1 alpha (CPT1α), the key rate-limiting enzyme in fatty acid oxidation, showed a significant 84% increase in expression in the GHM group (*p* = 0.0159) (Figure 6J,K). Additionally, the expression of hormone-sensitive lipase (HSL), a major enzyme involved in lipolysis, was notably elevated by 49% (*p* = 0.0375) in the skeletal muscle of DIO mice treated with a moderate dose of GB (Figure 6J–L). In summary, mid- and high-dose GB exhibited the most beneficial effects related to molecular regulatory mechanisms in the skeletal muscle mitochondria of DIO mice. Overall, AMPK activation and its downstream effects, such as mitochondrial biogenesis and FAO, are responsible for the reduction in muscular fat in HFD-induced obese mice.

## 4. Discussion

This study demonstrates that GB acts as a potent nutraceutical AMPK activator, modulating downstream targets that regulate skeletal muscle function in DIO mice. GB alleviated the clinical signs of DIO-inducible metabolic complications, including glucose intolerance and muscle weakness, partially by reducing muscle fat accumulation and activating AMPK. These beneficial effects may be associated with increased mitochondrial biogenesis and elevated fatty acid oxidation, both regulated through AMPK activation. Collectively, the results indicate that GB supplementation may offer notable metabolic and muscular advantages in the context of obesity by activating AMPK, thereby promoting mitochondrial biogenesis, enhancing fatty acid oxidation in skeletal muscle, and supporting broader metabolic homeostasis.

In a previous study, GB attenuated hyperglycaemia and hyperlipidaemia by inhibiting hepatic lipogenesis via AMPK activation in HFD-fed mice [43]. Additionally, GB increases muscle biogenesis by activating the Akt/mTOR/S6K pathway and prevents the activation of muscle atrophy induced by dexamethasone exposure [44]. However, our study presents conflicting results compared to previously reported data, particularly regarding discrepancies in in vivo experiments under HFD-induced conditions. Kim et al. (2022) demonstrated that a diet containing GB failed to protect against HFD-induced obesity after 12 weeks of dietary intervention [43]. This discrepancy may be due to differences in experimental conditions, such as feeding duration and the metabolic state of the mice. However, we also highlight the differences in the GB processing methods used in our study. Enzymatic hydrolysis is used to produce bioactive peptides with nutritional value [62]. Bioactive peptides, which are generated as a result of enzymatic hydrolysis, generally contain between 2 and 20 amino acid residues and are known to exert diverse biological activities, including antihypertensive, antioxidant, and antidiabetic properties [63]. Moreover, small peptides are absorbed more easily than larger molecular proteins and quickly reach target organs [64]. Metabolic syndrome-inhibitory peptides (e.g., KVEGDLK, YETGNGIK, AIGVGAIR, etc.) from a cricket protein fraction exhibited inhibitory effects on angiotensin-converting enzyme, pancreatic lipase, and α-glucosidase [65]. Fashakin et al. (2023) reported that antioxidant peptides (e.g., TEAPLNPK, EVGA, KLL, etc.) derived from GB protein hydrolysate demonstrated antioxidant activity [66]. Therefore, we speculate that the byproducts of the enzymatic hydrolysis of GB may exert further therapeutic effects on the skeletal muscles of DIO mice. Future peptidomics-based studies are planned to isolate and characterize novel bioactive peptides from GB hydrolysates.

Skeletal muscle is a metabolic organ that plays a crucial role in controlling systemic glucose homeostasis and energy expenditure [67]. Skeletal muscle is responsible for approximately 60–80% of glucose uptake following a meal in response to insulin, emphasizing its key role in insulin resistance associated with type 2 diabetes [7]. In individuals with obesity, an overload of lipids leads to the abnormal deposition of fat in non-adipose tissues, including skeletal muscle and the liver [8]. Ectopic fat accumulation potentially leads to systemic inflammation and impairs insulin signaling, thereby resulting in IR [4]. In this study, DIO mice exhibited decreased intramuscular fat deposition, enlarged muscle fiber cross-sectional area, and improved muscle strength (Figure 4C–I). These improvements suggest that GB not only ameliorates structural abnormalities in skeletal muscle but also enhances its functional capacity. While the majority of glucose-related parameters did not show statistically significant differences, blood glucose levels at 120 min after the OGTT were significantly reduced in the GB-treated groups compared to the H control group. Additionally, a general trend toward improved glucose tolerance was evident. This suggests that decreased lipid accumulation in muscle tissue may help reduce lipotoxicity and enhance insulin signaling, potentially contributing to the improved glucose tolerance observed. These results underscore the potential of GB as a nutraceutical candidate for supporting skeletal muscle health and overall metabolic regulation.

Despite its metabolic benefits, GB supplementation did not affect fat mass (Figure 2E–G) or adipocyte size (Figure 2H,I). Additionally, systemic lipid levels, including serum TG, TC, LDL-C, and HDL-C, remained unchanged after GB supplementation under our experimental conditions (Table 1). We propose three hypotheses to explain these findings. First, TG hydrolyzed in skeletal muscle may be released into the bloodstream, thereby mitigating changes in local fat accumulation without altering systemic lipid profiles. Second, GB may limit fatty acid entry into the muscle by modulating the expression of transporters responsible for fatty acid uptake in response to increased fatty acid utilization. This mechanism may prevent excessive fatty acid accumulation while maintaining energy balance. Ultimately, the influence of GB appears to be primarily confined to skeletal muscle, with limited effects on circulating lipid levels, which are largely controlled by various metabolic organs including the liver and adipose tissue. To further elucidate these mechanisms, future studies should be designed to investigate lipid metabolism-related markers in key metabolic organs, such as the liver and adipose tissue, and examine their correlation with the observed effects.

Considering that GB primarily improves skeletal muscle health, we focused on the regulatory effects of GB on skeletal muscle metabolism. Skeletal muscle plays a key role in systemic metabolism, partly through the release of various bioactive molecules known as myokines [68]. For instance, irisin, a myokine stimulated by PGC-1α activation, induces WAT browning and energy expenditure [69]. In our study, the mRNA expression of *Pgc-1α* exhibited an increasing trend in skeletal muscle of GB-treated mice compared to the HFD control (Figure 6A) and was also markedly upregulated in the palmitate-induced C2C12 myotubes following GB treatment. In addition, the mRNA expression of *Nrf1* and *Tfam* significantly increased in the GHM and GHM groups (Figure 6F), suggesting that GB may act as a stimulator of myokine secretion via PGC-1α-induced transcriptional programs. However, we did not observe any remarkable changes in the serum myokine levels, possibly because of the dosage and duration of GB administration. Therefore, the absence of marked changes in the WAT phenotype in the present study may be related to insufficient paracrine effects, with GB’s impact being primarily localized to the skeletal muscle.

AMPK activation plays a central role in metabolic regulation by promoting catabolism, such as energy expenditure and FAO, while inhibiting anabolic processes, including muscle protein synthesis, by suppressing mTOR [15,17]. A range of stimuli, including aerobic exercise, have been shown to activate AMPK, which subsequently stimulates PGC-1α and downstream regulators such as estrogen-related receptor alpha (ERRα), peroxisome proliferator-activated receptor gamma (PPARγ), as well as NRF1, and NRF2. Collectively, these factors drive mitochondrial biogenesis and enhance FAO, thereby facilitating metabolic adaptations [70,71]. Thus, AMPK is widely recognized as a promising molecular target for ergogenic interventions aimed at treating metabolic disorders [72]. In our experiments, GB partially lowered blood glucose levels (Figure 3), which is consistent with previous findings [43]. This response is likely driven by AMPK activation, which facilitates glucose uptake by promoting the movement of GLUT4 from intracellular storage vesicles to the muscle cell membrane (sarcolemma) [19]. Notably, GB administration significantly increased phosphorylated AMPK levels in the skeletal muscle following high-dose GB supplementation over the 16-week dietary intervention (Figure 4A,B). Additionally, the expression of *Glut4* was modestly upregulated by GB intervention (Figure 6G), which was in line with the increasing trend in blood glucose levels (Figure 3B,C). Furthermore, phosphorylated ACC levels were higher in the GHH group than in the N group (Figure 4A–C). The phosphorylation of AMPKα Thr172 activates the AMPK signaling pathway, leading to the phosphorylation of ACC Ser79. The phosphorylation of ACC inhibits fatty acid synthesis by reducing the conversion of acetyl-CoA to malonyl-CoA [11,12]. As malonyl-CoA is a potent inhibitor of CPT1α (which transports long-chain fatty acids into mitochondria for oxidation), reduced malonyl-CoA levels enhance fatty acid uptake and oxidation in skeletal muscle mitochondria [70]. In conclusion, GB, a novel muscular AMPK activator, may promote FAO while inhibiting lipid synthesis, contributing to partial improvement in glucose homeostasis.

Although GB acts as an AMPK activator in skeletal muscles, the precise mechanism by which it activates AMPK remains elusive. Metformin, an AMPK activator, indirectly activates AMPK by inhibiting complex I in the mitochondrial respiratory chain, leading to an increase in AMP/ATP ratio [15]. In contrast, the direct AMPK activator AICAR enters cells via AMP transporters and is phosphorylated by adenosine kinase, thus generating AMP-mimetic AICAR monophosphate [23]. Therefore, further in-depth investigations are needed to determine whether GB directly targets these kinases to activate AMPK or employs an alternative indirect mechanism, such as altering cellular energy status.

Given the significant reduction in muscular lipid content observed in DIO mice supplemented with GB, we investigated whether this effect could be attributed to increased FAO of ectopic fat in the skeletal muscle by analyzing the expression of AMPK downstream genes. In our study, GB upregulated key transcription factors related to mitochondrial biogenesis and function (Figure 6A–F), mirroring adaptations typically induced by endurance exercise [72]. PGC-1α, a key regulator of mitochondrial function, responds to environmental and intracellular cues and is tightly linked to AMPK. Upon AMPK phosphorylation, PGC-1α activates nuclear respiratory factors (NRF-1/2) [73], which in turn promote the transcription and expression of TFAM, which facilitates mtDNA transcription and replication, ultimately driving mitochondrial biogenesis [74]. In this study, 16 weeks of GB administration activated the gene expression of *Pgc-1α* and subsequently stimulated the downstream cascade, involving *Nrf1/2* and *Tfam* (Figure 6A–D). Moreover, *Nampt* gene expression was upregulated in the mid- and high-dose of GB feeding groups, suggesting that the effects of GB may be mediated through a sirtuin-dependent mechanism in skeletal muscle (Figure 6E). Furthermore, GB reversed the aggravating effects of the HFD on the expression of atrogenes, indicating that GB supplementation may not induce inflammation or muscle atrophy (Figure 6H). Interestingly, a high dose of GB supplementation resulted in lower transcriptional activity than a moderate dose, as seen in the expression of *Nrf1*, *Nrf2*, and *Ucp3*, suggesting that these effects may be saturated at a higher dose. This may be due to receptor desensitization or the activation of feedback regulatory pathways that counteract the effects of GB. Despite limitations in transcriptional regulation, the observed improvements in muscle health at the high dose suggest that GB may exert beneficial physiological effects without evident adverse effects, as reflected by unchanged liver and kidney weights (Figure S1). Additionally, lipolytic enzymes (e.g., HSL and LPL) may hydrolyze ectopic fat, releasing free fatty acids (FFAs) into the systemic bloodstream for ATP production [49]. These FFAs, released by lipolysis, can be further oxidized in the mitochondria, a process facilitated by the CPT1α-mediated transport of FFAs into mitochondria [75]. Our results showed that GB increased the expression of lipolytic enzymes (Figure 6I–L) without altering blood lipid levels (Table 1). Furthermore, GB significantly increased the protein expression of CPT1α in skeletal muscle (Figure 6K).

Edible insects contain numerous bioactive compounds, including various types of polyphenols. Insect-embedded polyphenols can be sub-classified into phenolic acids, coumarins, lignins, tannins, and flavonoids (e.g., flavones, chalcones, isoflavones, aurones, and flavanols) [36,37]. These components exhibit diverse biological activities, such as antioxidant capacity and anti-inflammatory effects [76], making them beneficial for robust skeletal muscle health [77]. However, a limitation of our study is that we could not identify the active compounds in GB. Nevertheless, based on our findings and previous literature, we speculate that GB may contain bioactive compounds with potential benefits for patients with obesity and diabetes [34,36]. Notably, GB has been suggested to be rich in arginine, and its supplementation has been associated with reduced adiposity, stimulated mitochondrial biogenesis, and an increased expression of genes that promote whole-body oxidation of energy substrates in diet-induced obese rodents [78]. Furthermore, GB contains an abundance of polyphenols, particularly flavonoids and phenolic acids. Resveratrol is a bioactive polyphenol that stimulates AMPK and improves mitochondrial function in vitro and in vivo [26,73,74]. The results of this study suggest that GB share similar bioactivities, potentially mimicking the effects of resveratrol [79]. However, further studies are needed to elucidate the underlying mechanisms in more detail. Our findings provide valuable insights into the functional properties of edible insects, including GB’s ability to enhance mitochondrial function and antioxidant capacity [80], potentially influencing lipodomic profiles. These results highlight the potential applications of GB as a nutraceutical agent for the prevention or management of metabolic complications, particularly within the context of a sustainable, green pharmaceutical industry.

## 5. Conclusions

Our results confirmed that GB effectively mitigated DIO-associated metabolic complications, such as glucose intolerance and ectopic fat accumulation. By reducing ectopic fat deposition in skeletal muscle, GB supplementation enhanced muscle health in DIO mice, likely by stimulating mitochondrial biogenesis and promoting the catabolism of ectopic fat within the muscles. The attenuation of ectopic fat accumulation by GB supplementation may be facilitated by increased FAO in skeletal muscle, potentially through post-translational modifications of key metabolic regulators such as AMPK and ACC. These findings suggest that GB may serve as a promising preventive and/or therapeutic strategy for improving metabolic health in obesity-related conditions such as sarcopenia.

## Figures and Tables

**Table 1:** Serum biochemical values in DIO mice.

Parameter	N	H	GHL	GHM	GHH
TG (mmol/L)	3.22 ± 0.28 ^a^	3.79 ± 0.50 ^a^	3.34 ± 0.25 ^a^	2.99 ± 0.48 ^a^	3.49 ± 0.38 ^a^
TC (mmol/L)	2.22 ± 0.58 ^a^	3.83 ± 0.58 ^b^	5.24 ± 0.47 ^b^	5.31 ± 0.37 ^b^	5.74 ± 0.25 ^b^
LDL-C (mmol/L)	0.97 ± 0.35 ^a^	2.27 ± 0.35 ^b^	2.87 ± 0.25 ^b^	3.04 ± 0.23 ^b^	3.14 ± 0.21 ^b^
HDL-C (mmol/L)	0.60 ± 0.03 ^a^	1.23 ± 0.12 ^b^	1.71 ± 0.17 ^b^	1.37 ± 0.07 ^b^	1.71 ± 0.13 ^b^
AST (IU/L)	47.62 ± 3.9 ^a^	51.94 ± 1.03 ^a^	57.04 ± 4.54 ^a^	42.25 ± 2.47 ^a^	37.63 ± 2.27 ^a^
ALT (IU/L)	5.86 ± 0.76 ^a^	20.89 ± 4.80 ^b^	17.54 ± 4.36 ^ab^	18.87 ± 3.52 ^b^	15.14 ± 4.53 ^ab^

Data are means ± SEM (*n* = 7–11). Values containing the same letter are not significantly different (*p* < 0.05). ALT, alanine aminotransferase; AST, aspartate aminotransferase; GHL, HFD + 100 mg/kg GB; GHM, HFD + 200 mg/kg GB; H, high-fat diet group; HDL-C, high-density lipoprotein cholesterol; HFD, high-fat diet; HFD + 400 mg/kg; IU, international unit; LDL-C, low-density lipoprotein cholesterol; N, normal diet group; TC, total cholesterol; TG, triglyceride.

## Data Availability

The original contributions presented in this study are included in the article/Supplementary Materials. Further inquiries can be directed to the corresponding authors.

## Supplementary Materials

The following supporting information can be downloaded at: https://www.mdpi.com/article/10.3390/nu17121990/s1, Table S1: Primers for qRT-PCR; Table S2: List of antibodies for western blot; Figure S1: Organ weight-to-body weight ratios.

## Abbreviations

36B4	Ribosomal protein lateral stalk subunit P0
AICAR	5-Aminoimidazole-4-carboxamide ribonucleotide
ACC	Acetyl-CoA carboxylase
ALT	Alanine aminotransferase
AMPK	AMP-activated protein kinase
ANOVA	One-way analysis of variance
ART	Aligned rank transform
AST	Aspartate aminotransferase
AUC	Area under the curve
CPT1α	Carnitine palmitoyltransferase 1 alpha
CSA	Cross-sectional area
DIO	Diet-induced obesity
EAT	epididymal white adipose tissue
ELISA	Enzyme-linked immunosorbent assay
ERRα	Estrogen-related receptor alpha
Fbxo32	F-box protein 32
FAO	Fatty acid oxidation
FFAs	Free fatty acids
GA	gastrocnemius
GB	*Gryllus bimaculatus*
GHL	HFD + 100 mg/kg/day GB group
GHM	HFD + 200 mg/kg/day GB group
GHH	HFD + 400 mg/kg/day GB group
GLUT4	Glucose transporter type 4
H	High-fat diet group
HDL-C	High-density lipoprotein cholesterol
H&E	Hematoxylin and eosin
HFD	High-fat diet
HOMA-IR	Serum homeostasis model assessment-estimated IR
HSL	Hormone sensitive lipase
IR	Insulin resistance
LDL-C	Low-density lipoprotein cholesterol
LPL	Lipoprotein lipase
mTOR	Mammalian target of rapamycin
N	Normal diet group
NAMPT	Nicotinamide phosphoribosyltransferase
NBF	Neutral-buffered formalin
ND	Normal diet
NRF1	Nuclear respiratory factor 1
NRF2	Nuclear respiratory factor 2
OGTT	Oral glucose tolerance test
PGC-1α	Peroxisome proliferator-activated receptor gamma coactivator-1 alpha
PPARγ	Peroxisome proliferator-activated receptor gamma
RAT	Retroperitoneal white adipose tissue
SEM	Standard error of the mean
SIRT1	Sirtuin 1
SQT	Subcutaneous white adipose tissue
T2D	Type 2 diabetes mellitus
TC	Total cholesterol
TG	Triglycerides
TFAM	Mitochondrial transcription factor A
UCP3	Uncoupling protein 3
WAT	White adipose tissue
