# Supplementary material for: Gryllus bimaculatus Hydrolysate Ameliorates Obesity-Induced Muscle Atrophy by Activating Skeletal Muscle AMPK in Mice
Source: Nutrients. 2025 Jun 12;17(12):1990. doi: 10.3390/nu17121990 (PMC12196165; doi:10.3390/nu17121990)
Supplement: Supplementary file 1 [file nutrients-17-01990-s001.zip › nutrients-3664258-supplementary.pdf]

Table S1. Primers for qRT-PCR

Primer	Sequences (5' – 3')
Pgc-1α-F	GAATCAAGCCACTACAGACACCG
Pgc-1α-R	CATCCCTCTTGAGCCTTTCGTG
Nrf1-F	GTCTGGATGGTCATTTCACCGC
Nrf1-R	GGCAACAGTAGCCACATTGGCT
Nrf2-F	CAGCATAGAGCAGGACATGGAG
Nrf2-R	GAACAGCGGTAGTATCAGCCAG
Tfam-F	CGAATCCTATCATCTTTAGCAAGC
Tfam-R	GAGGCAAAGGATGATTCGGCTC
Nampt-F	GGCACCACTAATCATCAGACCTG
Nampt-R	AAGGTGGCAGCAACTTGTAGCC
Ucp3-F	CAACTGTGCTGAGATGGTGACC
Ucp3-R	TGGCACAGAAGCCAGCTCCAAA
Glut4-F	GGTGTGGTCAATACGGTCTTCAC
Glut4-R	AGCAGAGCCACGGTCATCAAGA
Fbxo32-F	TCTTTTGGGCGATGCCACTCAG
Fbxo32-R	CTTCTCGACTGCCATCCTGGAT
Lpl-F	CGAGCGCTCCATTCATCTCT
Lpl-R	GGAGTTGCACCTGTATGCCT
36b4-F	GCTTCGTGTTCACCAAGGAGGA
36b4-R	GTCCTAGACCAGTGTTCTGAGC

Abbreviations: *36b4*, Ribosomal Protein Lateral Stalk Subunit P0; *Fbxo32*, F-box protein 32; F, Forword; *Glut4*, Glucose transporter type 4; *Lpl*, Lipoprotein lipase; *Nampt*, Nicotinamide phosphoribosyltransferase; *Nrf1*, Nuclear respiratory factor 1; *Nrf2*, Nuclear respiratory factor 2; *Pgc-1α*, Peroxisome proliferator-activated receptor gamma coactivator 1-alpha; R, Reverse; *Tfam*, Mitochondrial transcription factor A; *Ucp3*, Uncoupling protein 3.

Table S2. List of antibodies for western blot

Antibodies	Company	Cat. No.	Dilution
Phospho-AMPKα (Thr172)	Cell Signaling	2535	1:1000
AMPKα	Cell Signaling	2532	1:1000
Phospho-Acetyl-CoA Carboxylase (Ser79)	Cell Signaling	3661	1:1000
Acetyl-CoA Carboxylase	Cell Signaling	3661	1:1000
CPTI	Santa Cruz	Sc-393070	1:200
HSL	Cell Signaling	18381	1:1000
β-actin	Santa Cruz	Sc-47778	1:500
Anti-mouse IgG, HRP-linked Antibody	Cell Signaling	7076	1:2000
Anti-rabbit IgG, HRP-linked Antibody	Cell Signaling	7074	1:2000

Abbreviations: ACC, Acetyl-CoA carboxylase; AMPK, 5' adenosine monophosphate-activated protein kinase; CPTI, Carnitine palmitoyltransferase I; HRP, Horseradish peroxidase; HSL, Hormone-sensitive lipase; Ser, Serine; Thr, Threonine.

Figure S1. Organ weight-to-body weight ratios

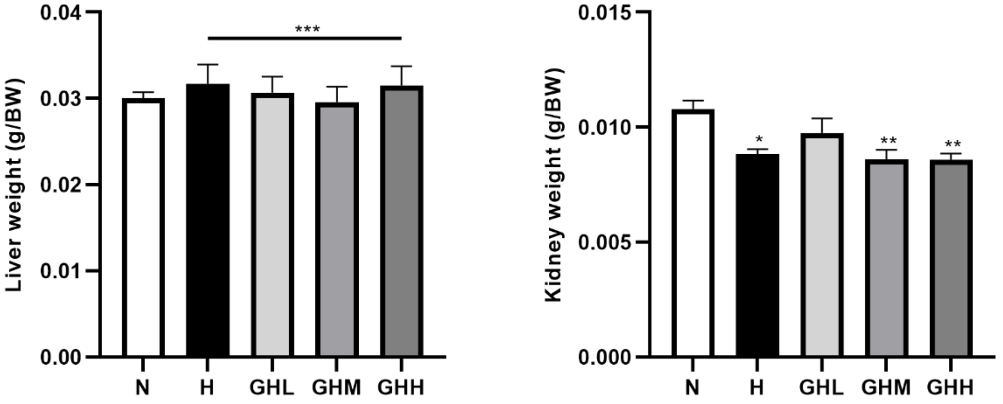

Figure S1. Effects of GB on organ weight-to-body weight ratios. Data represent the means ± SEM. * < 0.05, ** < 0.01, *** < 0.001 versus N group. BW, body weight; GB, *Gryllus bimaculatus*; GHH, HFD + 400 mg/kg; GHL, HFD + 100 mg/kg GB; GHM, HFD + 200 mg/kg GB; H, high-fat diet group; HFD, high-fat diet; N, normal diet group.
